# Deciduous Mandibular Second Molar with Supernumerary Roots and Root Canals Associated with Missing Mandibular Permanent Premolar

**DOI:** 10.5005/jp-journals-10005-1104

**Published:** 2010-04-15

**Authors:** Vivek Rana, Shabina Shafi, Natasha Gambhir, Usha Rehani

**Affiliations:** 1 Reader, Department of Pedodontics, Subharti Dental College, Meerut, Uttar Pradesh, India; 2 Lecturer, Department of Pedodontics, Subharti Dental College, Meerut, Uttar Pradesh, India; 3 Ex-Professor, Department of Pedodontics, Subharti Dental College, Meerut, Uttar Pradesh, India

**Keywords:** Supernumerary roots, Additional roots, Root canals, Deciduous teeth.

## Abstract

Morphological variations like additional roots and root canals in human deciduous dentition are rare. Knowledge of the morphology, variation of root and root canals of deciduous teeth are useful for successful endodontic treatment and exodontia. Presented here is a case report of the supernumerary roots and additional root canals of deciduous mandibular second molar (85) with congenitally bilateral missing of mandibular permanent second premolar (35 and 45) tooth bud.

## INTRODUCTION

An accurate diagnosis of the morphology of the root canal system is a prerequisite for successful root canal treatment.^1^ Frequently, root canals are left untreated because the clinicians fail to identify their presence, particularly in teeth that have an anatomical variations or additional root canals.^2^ Supernumerary root is a developmental condition and may involve any tooth.

The incidence of occurrence of three roots in deciduous second molar has been recorded to be 27.8%.^3^ Although any tooth may be congenitally missing, there is a tendency for certain teeth to be missing more frequently than others. Studies on the frequency of missing second premolar have shown this tooth to be congenitally absent in as many as 47.3% of all subjects examined.^4^

This report describes the presence of supernumerary root and additional root canals in deciduous mandibular second molar with congenitally missing mandibular second premolar tooth bud bilaterally.

## CASE REPORT

**Fig. 1:**
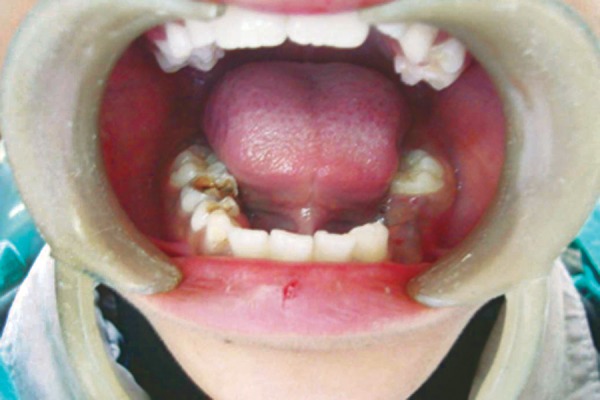
Preoperative view

**Fig. 2:**
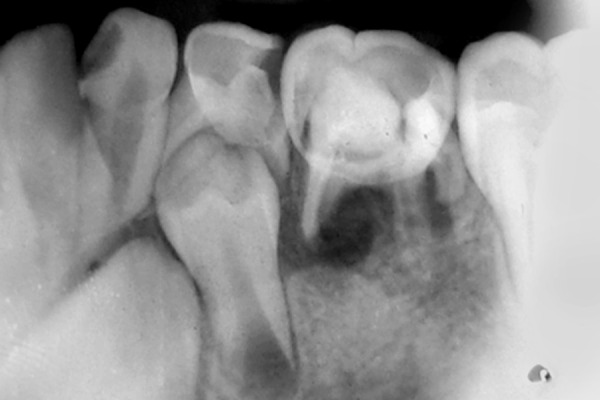
IOPA X-ray showing 85 and missing 45

**Fig. 3:**
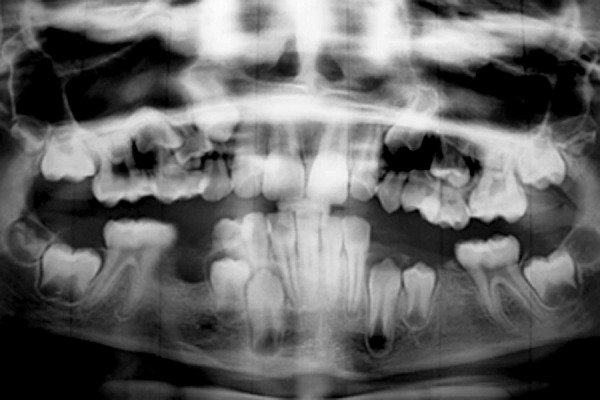
OPG showing missing 35 and 45

**Fig. 4:**
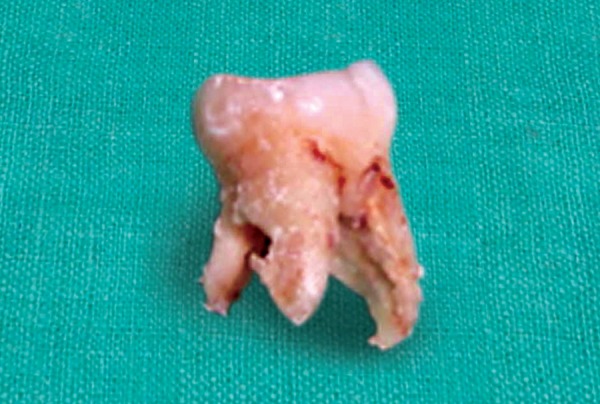
Extraction of 85 with three roots

**Fig. 5:**
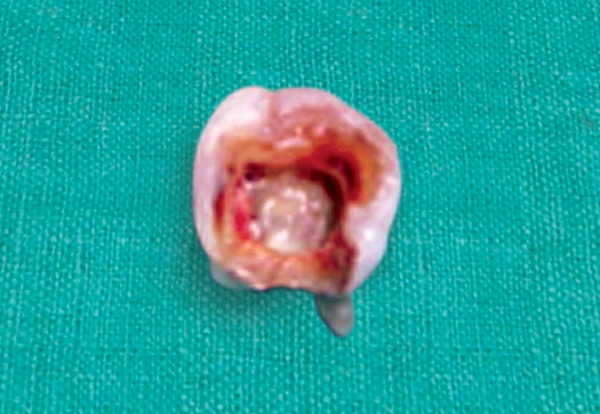
Access cavity preparation done *in vitro*

**Figs 6A and B:**
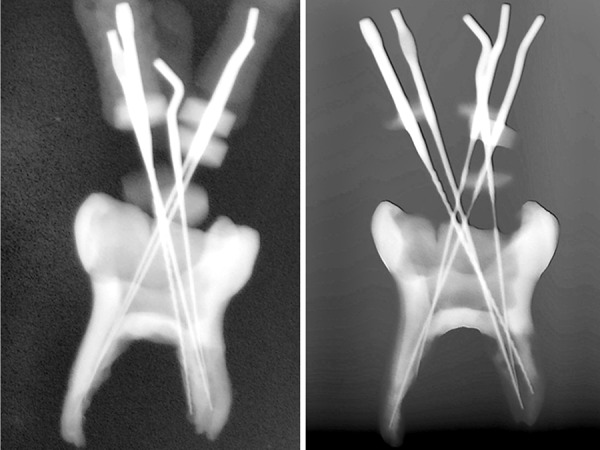
Radiographic view of working length with 5 root canals *in vitro*

**Fig. 7:**
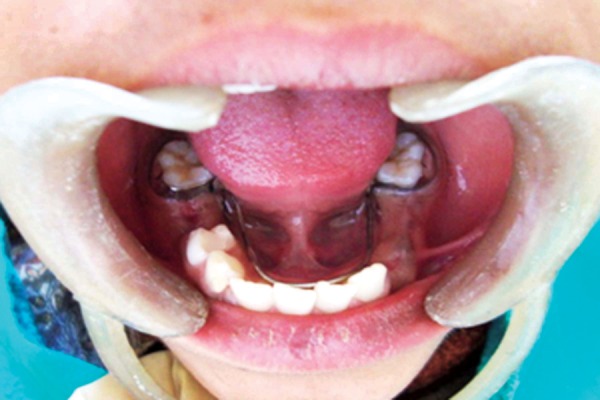
Lingual arch space maintainer

A 10-year-old male patient reported to the Department of Pedodontics of the Subharti Dental College, Meerut, India with complaint of decayed teeth in lower right region and desired to have it extracted. The patient had no systemic disorder. Clinical evaluation revealed carious lesions associated with mandibular deciduous canine, first and second molar (83, 84, 85) (Fig. 1). Further evaluation of the opposite side revealed missing mandibular deciduous canine, first and second molar (73, 74, 75), which he got extracted by a local dentist. A preoperative periapical radiograph of 85 confirmed the presence of extensive carious lesion with periapical abscess. Also, a possible anatomic variation was suspected in relation to 85. To further confirm the possible variation, IOPA radiograph was taken in different angula-tions which confirm the presence of three roots, that is two mesial and one distal and also with missing premolar (45) (Fig. 2). For further confirmation of any other missing tooth, OPG was taken and revealed missing mandibular second premolar (35) (Fig. 3). The decayed mandibular deciduous second molar (85) was extracted under local anesthetic because of poor prognosis (Fig. 4). On extracted tooth, access opening was made (Fig. 5), canals were explored with size 10 H file resulting in clinical and radiographic confirmation of 5 canals (3 buccal and 2 distal) (Figs 6A and B). The postextraction instructions were given to the patient and was recalled again for impression, and space analysis was done. In order to avoid space loss, lingual arch space maintainer was given (Fig.7). Mandibular deciduous canine and first molar (83 and 84) were restored with type II GIC cement.

Patient is kept under further follow-up so that later, for esthetic and functional reasons, lingual arch is replaced by removable partial denture due to missing mandibular premolars bilaterally.

## DISCUSSION

The morphology of root canals in deciduous teeth usually leads to complications in root canal therapy. Endodontic procedures for the treatment of deciduous teeth are indicated if the canals are accessible and there is an evidence of essentially normal supporting bone.^5^ In deciduous teeth, intimate anatomical relation of the pulp to the periodontal tissues via accessory canals result in concomitant pulpal periodontal breakdown. Deciduous mandibular second molar usually have two root (mesial and distal), but variations up to three roots have been reported.^6,7^ Deciduous mandibular molar have three root canals, viz. mesiobuccal, mesiolingual and distal canal. Accessory root canals in deciduous teeth were observed by Simpson, Skillen and Winter.^8-10^ According to Sarkar and Rao, prevalence of accessory canal in deciduous molar was less.^11^ Naser et al studied that all deciduous mandibular second molar have four canals: Mesiobuccal, mesiolingual, distobuccal and distolingual.^12^ In our case report, we found one accessory root, viz mesiolingual in deciduous mandibular second molar. *In vitro* access, opening of extracted deciduous mandibular second molar, we found five root canals. One additional root canal was found in mesiobuccal root and other in distal root.

Hypodontia is lack of development of one or more teeth. Although, the etiology of a single missing tooth is unknown. A familial tendency for this defect is present in many instances. Prevention of space, present in oral cavity due to missing teeth is very important because it can result in drifting of adjacent, opposing teeth and finally cause malocclusion.

## CONCLUSION

The phenomenon of supernumerary roots and root canals in deciduous molar with missing permanent premolar is of considerable significance in pedodontics. Accurate knowledge of root and root canal morphology helps in endodontic treatment and extraction of teeth. Additional root may be broken off during extraction, if unrecognized and allowed to remain in the alveolus, may be the source of future infection.
